# White supremacy and the racial logic of the global preventing and countering violent extremism agenda

**DOI:** 10.1080/01436597.2024.2370358

**Published:** 2024-07-11

**Authors:** Elizabeth Mesok, Nora Naji, Darja Schildknecht

**Affiliations:** Department of Social Sciences, University of Basel, Basel, Switzerland

**Keywords:** Counterterrorism, white supremacy, violent extremism, peacebuilding

## Abstract

This article analyses what the disavowal of abject forms of white supremacy reveals about the racial logic of the global preventing and countering violent extremism (P/CVE) agenda. We argue that the global P/CVE agenda is built on racialised concepts such as prevention, radicalisation and community – concepts that render it incommensurate with the newly identified problem of white supremacist violent extremism or domestic terrorism. Through analysis of interviews with experts and practitioners working within the broad field of P/CVE, we discursively analyse how the enmeshment of the agenda within the development and peacebuilding space exposes the agenda’s primary intent to manage presumably ungovernable populations in or from the so-called Global South. Taking the theoretical insights culled from textual analysis of practitioner interviews, we then consider the inclusion of right-wing extremism, and specifically white supremacy, within Western states’ domestic P/CVE agendas, primarily in the US. Our argument – that the move to consider far-right extremism within domestic CVE policy reveals rather than disrupts the P/CVE agenda’s racist foundations and intentions – contributes to a growing body of research that insists on attending to race, racialisation and racism within security studies and international relations, and which includes an emphasis on whiteness as an organising principle.

## Introduction

In Spring 2021, US President Joe Biden announced a new strategy to address the ‘persistent and evolving’ threat of domestic terrorism. Of the range of ideologies that stand to harm the US, ‘racially or ethnically motivated violent extremists (principally those who promote the superiority of the white race) and militia violent extremists are assessed as presenting the most persistent and lethal threats’ (United States 2021, 6). Since the establishment of the Department of Homeland Security (DHS) in 2003, the US security and intelligence community has spent more than two decades preventing terrorist attacks within the US, focused on the threat posed by foreign terrorist organisations (FTOs) and the so-called ‘home-grown violent extremists’ who are working to implement their objectives. Despite the US’s long history of white supremacist violence, since the terror attacks on 11 September 2001, actors named ‘terrorists’ and ‘violent extremists’, both within and beyond the borders of the US, have overwhelmingly been conceptualised as non-white men. The violence posed by white supremacists, for example, has remained at the periphery of the concerns of the security and intelligence community. The DHS, correspondingly, has paid little attention to the terror wrought by racism and white supremacism – that is, until Donald Trump incited insurrectionists to attack the Capitol on 6 January 2021.

Previously, the US’s anti-terror infrastructure, sanctioned by the US Patriot Act and consolidated within the DHS, primarily targeted Muslim and Arab American communities as the intended subjects of its domestic countering violent extremism (CVE) agenda. Over the last two years, however, policies have emerged that attempt to extend the US domestic CVE agenda to address violence enacted by white supremacists or so-called ‘far-right extremists’. It might seem obvious that a Democratic president, inaugurated mere weeks after far-right extremists stormed the seat of the federal government’s legislative branch in an attempt to challenge the results of the 2020 presidential election, would need to explicitly repudiate the racism that Donald Trump successfully mainstreamed during his tenure. To be sure, Biden’s condemnation of white supremacy and his identification of far-right extremists as the most urgent threat facing the US is politically savvy. However, the naming of far-right extremism, and white supremacy in particular, as *the* terrorist ideology threatening the US does more than just win Biden points with progressives: it disavows white supremacy as antithetical to the principles of liberal democracy and both justifies and expands the security state, which continues to disproportionately harm communities of colour.

This article analyses what the disavowal of abject – or demonstrably violent and unacceptable – forms of white supremacy in the US reveals about the racial logic of the global preventing and countering violent extremism (P/CVE) agenda. Although we were initially inspired by the Biden administration’s new approach to domestic terrorism, the subject of our article shifted to focus on the global P/CVE agenda – the non-kinetic complement to the global war on terror that has been nearly exclusively implemented in Muslim communities across the world.^1^ We argue that the global P/CVE agenda is built on racialised ­concepts such as prevention, radicalisation and community – concepts that render it incommensurate with the newly identified problem of white supremacist violent extremism or domestic terrorism. Our argument is built in two parts: the first, based on interviews with 14 experts and practitioners working in the broad field known as P/CVE, argues that the enmeshment of the agenda within the development and peacebuilding space exposes that the global P/CVE agenda’s primary intent is to manage supposedly unstable or ungovernable spaces or populations. In other words, the global P/CVE agenda was designed to discipline Muslim communities in the Global North and Muslim states and/or populations in the Global South, in order to ensure the security of the Global North.^2^ This occurs through what we, inspired by the analysis of one practitioner interview, call the ‘thingification’ of the P/CVE agenda and of violent extremism itself – the ways in which the agenda has taken abstract, nebulous conceptions, actions and relations, and rendered them ‘a thing’ upon which the security sector can act or a tool that the security sector can wield. This thingification means that the P/CVE agenda’s location within the nexus of peace–development–security is able to absorb the intention or political will of its implementers, reducing its legitimacy and efficacy as a mode of violence prevention. Further, discursive analysis of the interviews reveals that the concept of radicalisation is inherently related to Muslim populations and the prevention of Islamist extremism. From here, we argue that the P/CVE agendas’ design to prevent radicalisation depends upon particular conceptualizations of ‘the community’, and mirrors civil counterinsurgency practices to infiltrate and inoculate populations against insurgencies. Such analysis of interviews with P/CVE experts and practitioners contributes to recent scholarship that considers the discursive and racialised construction of counterterrorism and P/CVE within the United Nations (UN) (Martini 2021). While we recognise that 14 interviews are not representative of the broad community of practice that comprises P/CVE, we nevertheless argue the interviews contain traces of a racial logic that informs how ‘community’, ‘radicalisation’ and ‘prevention’ are conceptualised.

The second part of the article takes the theoretical insights culled from analysis of the practitioner and expert interviews and applies it to a consideration of the inclusion of right-wing extremism, and specifically white supremacy, within Western states’ domestic P/CVE agendas, primarily in the US. Alongside discursive analysis of US policy documents, we engage with theories of disavowal built from postcolonial scholars’ engagement with psychoanalytic theory. While such scholarship has generally theorised disavowal as it relates to mimicry and the identity of the colonised subject, we extend the theory here to consider the disavowal of abject forms of white supremacy. The disavowal of white supremacy within counterterrorism and P/CVE discourse is not synonymous with its repudiation or rejection. Rather, as postcolonial theorist Homi Bhabha writes, ‘disavowal is the *tolerance* of contradictory belief – and the subject’s splitting – made possible by a defensive, compromise formation’ (2015, 13, emphasis in original). What happens when white supremacy is disavowed by the very power structures that it built? We analyse the disavowal of abject forms of white supremacy within policy documents, as it relates to conceptualisations of violent extremism and terrorism, and consider what this means for the international counterterrorism architecture.

The research for this article comes out of a larger project focused on the development and implementation of gendered P/CVE policy and practice across overlapping sectors, including multilateral development agencies, peacekeeping operations, civil society organisations, and military and state institutions. At the outset of this project, we conducted expert interviews in spring 2021 with P/CVE researchers and practitioners from international research institutions and the UN. The intention of these semi-structured interviews was to assess the current state of P/CVE policy and practice, particularly as it related to gender. What we did not necessarily anticipate, however, was how relevant discourses of white supremacy and the logic of whiteness would be in these interviews, both in their presence but also in their absence. This finding led us to further analyse US government policies related to P/CVE and in particular those that had incorporated white supremacy within a definition of violent extremism.

Our argument – that the move to consider far-right extremism, and white supremacy in particular, within domestic CVE policy reveals rather than disrupts the P/CVE agenda’s racist foundations and intentions – contributes to a growing body of research that insists on attending to race, racialisation and racism within security studies and international relations (Machold and Charrett 2021; Chandler and Chipato 2021; Sabaratnam 2020), and which includes an emphasis on whiteness as an organising principle (Guerra 2021). Making explicit the role of whiteness and white supremacy within the P/CVE agenda is a necessary complement to the scholarship on race and racism within P/CVE which has, with good reason, focused on anti-Blackness and Islamophobia embedded within and unleashed by the counterterrorism agenda (Meier 2024; Khan 2021; Kundnani 2015; Abu-Bakare 2020).

Indeed, the epistemological issue of what Alison Howell and Melanie Richter-Montpetit call the ‘disciplinary whiteness’ (2023) of international relations – including the sub-disciplines of critical security studies – has resulted in very few critical interrogations into P/CVE as both a racial and racialising structure. Laura Shepherd, however, has recently argued that P/CVE initiatives present gendered subjects as ‘race-neutral’ and further, that the ideology of white supremacy and the privileging of whiteness ‘infuse and inform structures and institutions of global governance, including those engaged with P/CVE …’ (2022, 736). Shepherd’s argument informs our reading practice, where we recognise that programming and policy within interviews were largely presented as ‘race-neutral’ and yet were clearly structured by ideologies of white supremacy. Analysing P/CVE discourse for its foundational logic of whiteness, an ‘unmarked, unnamed, and non-racialized norm, taken for granted and therefore naturalized’ (Guerra 2021, 28), is thus a necessary first step to understanding the P/CVE agenda’s current pivot towards a disavowal of white supremacy.

## Targeting ‘the community’: the racial entanglements of P/CVE in peacebuilding and development

The P/CVE agenda emerged as a complement to the kinetic elements of the global war on terror. Justifying that the war on terror could not be won through policing, surveillance, military intervention and intelligence alone, the international community adopted a prevention-focused agenda designed to address the reasons why individuals join non-state armed groups and violent extremist or terrorist organisations in the first place. Whereas the early years of the global war on terror saw ‘the enemy’ as an irrational, despicable and inherently evil individual or group of individuals driven by religiosity, and who could not be reasoned with and must be destroyed (Martini 2021), this strategy of attrition warfare was eventually supplemented by a belief that ‘the enemy’ could also be stopped before they ever even came into being. With the adoption of the UN Plan of Action to Prevent Violent Extremism in 2016, ‘the community’ became yet another target of the global war on terror, albeit as a site of prevention rather than an object for destruction. However, recent attempts to adapt P/CVE programming for far-right, white supremacist movements suggest that the original meaning of ‘the community’ within the initial P/CVE framework was always meant to target racialised communities, particularly the Muslim community.

The emphasis on infiltrating and protecting communities – what one practitioner aptly described as a ‘ring of inoculation’ (Interview 6) – against the threat of violent extremism and terrorism mirrors pacification tactics used in civil counterinsurgency. The ‘hearts and minds’ element of P/CVE – the idea that violent extremism and terrorism cannot be defeated by sheer force but requires shifting the support of the population away from insurgent groups or, in this case, terrorist organisations – echoes the colonial logic of counterinsurgency tactics used as intertwining modes of governance and warfare (Kienscherf 2016). Recent scholarship has identified P/CVE as a ‘mode of civil counterinsurgency enacted through the peace–security–development nexus’ (Mesok 2022, 2) that coerces civil society organisations to participate in the security agenda through community policing and other ‘whole-of-­society’ programmes targeted to prevent violence. Making explicit how P/CVE functions as a mode of counterinsurgency builds upon scholarship such as Mandy Turner’s (2015) analysis of Western donor-led peacebuilding activities in Occupied Palestine as a form of counterinsurgency. As Turner argues, ‘explicitly relabeling peacebuilding as counterinsurgency’ offers a ‘deep grammar’ through which to analyse the seeming contradictions and paradoxes present within Western iterations of peacebuilding and development policy and practice. As Turner writes:

Modern counterinsurgency doctrine (COIN) is based on the idea that successfully securing a population and immunizing it against unrest requires serious and extensive strategies in the realm of governance, development and security. Western donor-led peacebuilding also focuses on these spheres – albeit with the proclaimed positive goal of developing mechanisms to avoid and/or reduce violent conflict and build a sustainable peace. (2015, 74)

Following Turner, in this section we discursively analyse the interviews with practitioners and experts on P/CVE with an eye towards the ‘deep grammar’: the racial and colonial logic of counterinsurgency reiterated by or mirrored in P/CVE. Our analysis of these practitioner and expert interviews is not meant to assign intention to the individuals; on the contrary, we understand the subjects as produced through and emblematic of larger institutional powers and thus read the interviews as evidence of a larger, global discourse grappling with the intentions and limitations of the P/CVE agenda.

### The triple nexus and the ‘thingification’ of P/CVE

Differentiated from terrorism, violent extremism is understood as a system of beliefs that drives individuals to commit violence against civilians (Schirch 2018). Rather than focus on preventing acts of terror, the intention behind P/CVE initiatives is to address the reason behind the behaviour – the process of radicalisation – to tackle the extremism that leads to violence in order to prevent it from occurring in the first place. Thus, the global P/CVE agenda emerged as a government-led framework intended to fund civil society organisations who are working towards violence prevention in their communities. P/CVE programming can have either a direct or an indirect relationship to violent extremism – this has resulted in the ‘rebranding’ of a wide array of activities formerly identified as either peacebuilding or development as P/CVE. Tracing the entanglement of P/CVE with Western-led peacebuilding and development agendas reveals foundational conceptualisations of who and what P/CVE was always intended to address.^3^

Over the last two decades, the peacebuilding–development nexus has undergone paradigmatic shifts, which include, among other transformations, a deeper and greater emphasis on the prevention of violent extremism through ‘whole-of-society responses’ (McCandless 2021). In addition, a new nexus has emerged: the humanitarian–development–peace nexus or the ‘triple nexus’. In February 2018, UN Secretary General António Guterres launched the UN Global Counter-Terrorism Coordination Compact, which positions P/CVE at the intersection of ‘the three pillars of work of the United Nations: peace and security, sustainable development, human rights and humanitarian affairs’ (UNOCT 2023). Ali,^4^ a security analyst and P/CVE practitioner, reflected on the difference of working before and after the emergence of the P/CVE agenda, which they saw as having brought the language and frameworks of development and peacebuilding into the security space:

It’s extraordinary to have gone from a world in which development officials and counterterrorism officials were never in the same meeting or, frankly, had the same vocabulary … [to] a world in which you have coordination bodies that are made up of both development, peacebuilding actors and security actors working in some level of coordination. (Interview 1)

The increasing overlap of development, peacebuilding and security ‘vocabulary’ within P/CVE spaces speaks to the location of P/CVE as the point of convergence for these previously distinct sectors.

The centrality of P/CVE within the triple nexus has proven challenging for some practitioners, however, as the approach one takes to the agenda is deeply informed by their own positionality within the nexus as well as their career training and experience. Jan, another practitioner, explained that this can lead to ‘a lot of animosity’ within the P/CVE ‘community of practice:’

I think where you enter into the longitudinal horizon matters … I think people from different backgrounds enter into the agenda with different agendas, but those who come from a harder securitised space [such as] counter-terrorism, law enforcement, intelligence and the military, see this very much as the soft side of their work. But people who enter this from humanitarian, development, local peacebuilding or a variety of other social sectors, mental health, physical wellbeing … they see this is a very securitised agenda. (Interview 2)

For Jan, one thing that has contributed to the ‘messiness’ of the topic is the ‘nebulous nature’ of the definition of ‘violent extremism’ and the difficulty of distinguishing it from other categories such as political violence. While the reduction of violence of any kind is always commensurate with peacebuilding objectives, the overemphasis on ‘violent extremism’ has exceptionalised it to the point of elusiveness. As Jan stated:

I don’t think P/CVE is actually a thing. I think it’s an organising principle for policymakers to try and see an aggregate outcome … It got thingified over about 15 years of bureaucratic movements to try and say this is a P/CVE-specific policy, this is a P/CVE-specific programme. But I think actually the vague shared priorities that P/CVE has always had with other agendas has meant that it’s never been a differentiated phenomenon, despite the fact that it’s had quite a bit of political will behind it. Because, ultimately, the social science on human behaviour or the political science on contextual factors or the concept behind international development and aid and assistance and the ways in which that gets done, all of these can’t be isolated from violent extremist organisations’ *raison d’être* and the way in which they are able to continue to attract people to their cause. (Interview 2)

These two quotes point to critical tensions within the P/CVE agenda. Even while the concept of violent extremism might be helpful in that it shifts the focus towards addressing underlying reasons why an individual might join an organisation or commit violence, the agenda that has been created to implement such violence prevention has been simultaneously oversignified and emptied of meaning, a loaded yet blank canvas onto which practitioners and policymakers can assign significance depending on their political will, their positionality, and even their personal understanding of the concept.

The ‘thingification’ of P/CVE offers a helpful analytical framework. The notion of thingification refers to the act of taking an abstract concept and treating it as if it were a material entity, immutable and concrete. While P/CVE has undoubtedly become what Andrea Cornwall has referred to as a ‘fuzzword’ – a buzzword within development practice that provides concepts ‘that can float free of concrete referents, to be filled with meaning by their users’ (2007, 474) – we find the concept of thingification particularly insightful as it moves beyond the discursive to include the material. In the Hegelian-Marxist tradition, thingification is understood as a synonym for reification, or ‘the transformation of human properties, relations, processes, actions, concepts, etc. into *res*, into things that act as pseudopersons, endowed with a life of their own’ (Vandenberghe 2015, 203). Theorising both violent extremism, which includes the actions and beliefs that lead to terrorism, and P/CVE as a policy framework that has been developed to respond to such actions and beliefs, as having been ‘thingified’ within the last decade requires recognising the interplay between the discursive realm and the materiality of development and security. Further, understanding both violent extremism and P/CVE as informed through processes of thingification forces an interrogation of the racialised ‘deep grammar’ that precedes ‘the thing’, the discursive ground that shapes the subjects of violent extremism into objects of P/CVE.

P/CVE’s situatedness at the intersection of the triple nexus requires an analysis of P/CVE’s capacity to absorb meaning, to be made into a ‘thing’ that has relevance to or that will benefit the donor or implementing actor. For instance, Sai, a project manager responsible for implementing P/CVE programming for a UN agency, explained that they frame their P/CVE initiatives as either peace or development, which allows them greater access to member states that might otherwise be reluctant to accept P/CVE, either due to their refusal to acknowledge the issue of violent extremism or even the government’s own collusion with such actors. In one context, Sai explained, they might use the language of sustaining peace, whereas in another they focus on building community resilience (Interview 3). Regardless, the focus is always on prevention. ‘Ultimately, PVE is a framework of conflict prevention’, Sai said (Interview 3). The location of P/CVE actors within the triple nexus determines the discourse that is used to both identify and analyse the problem. Ali, who has worked for both the DHS and the US Federal Bureau of Investigation (FBI), argued that the dispersion of development and peacebuilding discourse into the security community, for instance, was ‘one of the best things to have happened for our approach to counterterrorism’, given how it has shifted the attention of the security world towards macro-level, structural factors driving and enabling violent extremism (Interview 1). Considering the thingification of P/CVE within the peace–security–development nexus forces a recognition of how P/CVE gains meaning depending on where in the nexus the practitioners sit.

The thingification of P/CVE thus means that practitioners are able to manipulate and instrumentalise the discourse to their advantage, with clear material consequences. This is evident in how practitioners adjust their language, not only in the communities and contexts in which they implement programming, but also with donor states. One practitioner, Max, recounted that when working with the US, one has to use the language of CVE: ‘They want to have the “C” element, while actually you can do “P” in practice operationally and just say that you do the “C”. So, then they’re happy’ (Interview 4). Other practitioners were blunt about the utility offered by the vague or abstract quality of the P/CVE agenda. As Blake said:

I would define it just as a new brand that serves the purpose of fundraising for existing projects … If you see where the agenda has gone over the last few years, it’s really UNDP [the United Nations Development Programme] who has capitalised the most on this agenda without necessarily actually attempting to define it, or even defining it in the way they’ve acted on the ground. (Interview 5)

In a way, the thingification of violent extremism has resulted in a nebulous and capacious agenda that enables multilateral organisations, member states and civil society organisations to shapeshift, to strategically use the desired language in order to secure funding. What this risks, however, is the oversimplification of both the problem and the solution; or, as Jan called it, the ‘specificity trap’. As they explained, this can occur if ‘you get too focused on violent extremism and that ends up being the goal and you get really blind to the ecosystems and structure and dynamics and all these very dynamic moving pieces where VE [violent extremism] exists and where it emerges’ (Interview 2). Blake agreed that ‘the risk is that it puts a blanket on very complex issues. And so, it simplifies them and frames them under a very negative lens’ (Interview 5).

It is extremely difficult, if not impossible, to disentangle the discourse of the agenda and its implementation – or, in other words, how the agenda is used and what it means are intertwined. This is nowhere more evident than with the reports from Muslim communities, which interpret the global war on terror and the P/CVE agenda as an explicit war against Muslims. Interrogating the ‘deep grammar’ of P/CVE reveals a genealogy of racialised interventions across the development, peacebuilding and security spaces that have long replicated colonial logics. Functioning both as an empty signifier and a container for a broad range of interventions, the agenda’s emphasis on prevention gives insights into flawed and coercive interventions in Muslim communities under the premise of peace, development and humanitarian assistance.

### P/CVE and the reification of radicalisation

The P/CVE agenda emerged as a direct response to terrorism perpetrated by Islamist movements and organisations, further cementing the association of ‘terrorism’ and ‘violent extremism’ with ‘Muslim’. Jan, who has worked in the peacebuilding field for over two decades, stated:

Some of the earliest conceptions of P/CVE in the US government circles were seeking to truly understand the ideological underpinnings … of the violent extremist organisations that were the highest priority for the US government … pretty much Al-Qaeda and its affiliates, ISIS [the Islamic State of Iraq and Syria] and its franchisers, and the list went on. (Interview 2)

P/CVE’s emphasis on violent extremism and terrorist organisations that claim Islam as their cause or justification for violence has led to the exclusion of other violent organisations. As P/CVE scholar Mohammed Abu-Nimer writes:

While such programs exist, it is rare to identify or give wide media coverage and recognition to a program that addresses violent extremism … motivated by the Jewish settlers in the occupied Palestinian territories, white supremacist groups in the United States, Sri Lankan, and Myanmar Buddhism, or Indian Hinduism in Gujarat or Kashmir. (2018, 218)

The rhetorical and political emphasis on Islam in relationship to P/CVE can be described as, in Abu-Nimer’s words, the ‘Islamization of P/CVE’ (2018, 218). While Abu-Nimer believes that P/CVE can be ‘delinked’ from Islam and Muslim communities, we argue that the foundational mythologies upon which P/CVE was constructed present political and practical limits to such a decoupling.

The discourse of P/CVE – its deep grammar – is built upon and reproduces racialised understandings of radicalisation, violent extremism and terrorism. As P/CVE expert Blake explained, despite the precautions taken by the UN, P/CVE ‘still has been perceived as stigmatising Muslim populations and even certain populations within the Muslims, the Fulani, especially in the Sahel context’ (Interivew 5). Sascha, a P/CVE practitioner based in Western Europe, put it bluntly: ‘I think violent extremism has always been a code word for Islamic extremism’ (Interview 6). Marte, another P/CVE expert and practitioner, agreed and went on to explain the difficult conversations that took place in their organisation regarding the attempts to untangle the difference between political violence and violent extremism:

I think there is a sense that’s fairly widely shared that these are very political definitions and that the majority group gets to decide whether it’s political violence or violent extremism. And so, the othering of Muslims and Islamophobia, I think is a really clear dynamic in the P/CVE conversation. It’s why so many civil society groups have not wanted to take on that term, or that name, because … it’s so loaded with Islamophobia in some places; it has a heavy reputation, particularly among Muslim communities. (Interview 7)

The dominant association of P/CVE with Islamophobia and a ‘war on Muslims’ is due to the fact that the global P/CVE agenda – which has swallowed or ignored counter-extremism programming that existed prior to September 11 and which did not focus solely on Islamist extremism – has emerged squarely out of the global war on terror, which has targeted Muslim-majority states and communities the world over. The ‘political definitions’ and ‘heavy reputation’ of the P/CVE agenda must be contextualised within the greater global war on terror and, further, understood alongside the emphasis on pre-emption and prevention in theories of radicalisation.

For instance, the US’s supposed shift towards prevention in the global war on terror was not a radical break with the doctrine of pre-emptive war, but rather a continuation of it by other means (Stampnitzky 2013). In the years following the 11 September attacks, new security paradigms directed at the figure of the Islamist male foreign fighter as a ‘universal enemy’ emerged, producing alliances among Western states guided by the racialised logics of US empire (Li 2020; Bakali and Hafez 2022). The P/CVE agenda, as an extension of the global war on terror, embeds a logic of pre-emption that shapes the use of military interventions, unlawful detainments and expanded state power. Pre-emptive intervention in the form of preventing violent extremism entails psychology of terrorism approaches that focus on trajectories of radicalisation in order to understand what drives individuals to engage with terrorist or violent extremist organisations. However, once Western states realised that terrorism would not be defeated through brute military force, the focus shifted to understanding how terrorists are made, in order to prevent or disrupt that process. Arun Kundnani’s writing on the racialised assumptions inherent in the concept of radicalisation is worth quoting at length:

… the concept of radicalization inherited at birth a number of built-in, limiting assumptions: that those perpetrating terrorist violence are drawn from a larger pool of extremist sympathizers who share an Islamic theology that inspires their actions; that entry into this wider pool of extremists can be predicted by individual or group psychological or theological factors; and that knowledge of these factors could allow government policies that reduce the risk of terrorism. The study of radicalization, ostensibly a reflection on the cause of terrorism, is thus, in practice, limited to a much narrower question: why do some individual Muslims support an extremist interpretation of Islam that leads to violence? (2012, 5)

While the typology of radicalisation that Kundnani delineated has undergone revisions over the last few decades (see Silva 2018), the foundational concept as deployed within the global war on terror is indisputably centred on Muslim communities and what happens therewithin.

Interestingly, the emergence of the P/CVE agenda within the peacebuilding and development space has allowed for theories of radicalisation and approaches to violence, as Jan put it, to engage with a ‘humanist approach’ (Interview 2). In Jan’s words, P/CVE’s engagement with peacebuilding has shifted the focus to trying to understand:

why people join violent extremist organisations from a neuro-biological perspective, from a sociological and psychological perspective and try to be a little bit more humanist; the idea that people are not born terrorists and there are a variety of both personal and behavioural, group and structural ways in which people join violent groups and violent identities. (Interview 2)

This focus on a ‘humanist’ – or what we would more aptly term ‘human-centred’ – approach appears to offer a race-netural or colour-blind perspective to policy and programming, where individuals are ostensibly considered for their vulnerabilities, rather than condemned for them. Theoretically, this is what the inclusion of white supremacists into the categorisation of violent extremism is attempting: to build a human-centred peacebuilding approach that considers all individuals as similarly vulnerable to ideologies of hatred and violence. The idea that ‘people are not born terrorists’ underscores the agency of individuals to withstand their environments rather than succumb to non-state armed groups or violent extremist organisations. However, this human-centred approach does not necessarily dislodge racialised notions of radicalisation so much as it obfuscates racialised markers by emphasising the role of poverty and marginalisation in P/CVE interventions. Within a human-centred approach, all individuals ostensibly contain the agency to overcome their circusmtances; their inability to do so individualises terrorism and violent extremism even as such programming attempts to cast a wider understanding of the phenomena.

### P/CVE, counterinsurgency and the community

Community engagement is at the centre of P/CVE: community actors must either be or get engaged, whether to build social cohesion, to disseminate counternarratives, to foster resilience or to receive and enact development initiatives. Sai explained that communities are a key element and ‘making sure that communities are more resilient to violent extremism’ is a critical component of P/CVE (Interview 3). Alice Martini describes this process as:

securit[ising] the Muslim community, the Other living in the inside, rendering it a place of intervention for countering and preventing extremism. […] [W]hile terrorists/extremists were constructed as irrational and evil foes, Muslim communities were securitised through their discursive association to this violence. (2021, 147)

Thus, it is by virtue of being Muslim that Muslim communities are constructed as ‘at risk of becoming risky’ (Heath-Kelly 2013, 397). Part of building resilience is strengthening what is known in the development field as ‘social cohesion’, which the UNDP defines as ‘the extent of trust in government and within society and the willingness to participate collectively toward a shared vision of sustainable peace and common development goals’ (UNDP 2020, 16). Building social cohesion through fostering resilience and community engagement is considered more economically efficient than the violent conflict that would ensue if such measures are not taken. As Sascha explained:

If it’s $1.3 million for every hellfire missile to take out a $3,000, 28-er Subaru that has an IED [Improvised Explosive Device] in it or something – the comparison between PVE [Preventing Violent Extremism] programming going in and doing some … support and some mentoring and setting up peace clubs and digital clubs in schools in this particular radius, it’s absolutely peanuts. (Interview 6)

Although a rather crass comparison, and not necessarily accurate if one considers the war economies that benefit when terrorism is *not* prevented, this statement reveals the ways in which community relations have themselves become thingified, or turned into a commodity for the P/CVE agenda.

Ultimately, the emphasis on the community within P/CVE mirrors the tactics of civil counterinsurgency: building trust between security forces and the population and disciplining populations into self-governing, self-surveilling entities. Practitioners noted the commonalities between counterinsurgency and P/CVE, with Marte reflecting on the recognition by security forces ‘that community engagement is important and [also] this whole “hearts and minds” framework’ (Interview 7). The recognition that P/CVE tends to mirror some of the objectives of counterinsurgency, such as pacification or intelligence gathering, further reveal the racialisation of the imagined ‘community’ at the heart of the P/CVE agenda. Marte’s reflection on this tension is instructive:

The whole idea of P/CVE, it was similar to counterinsurgency in the sense of trying to separate – counterinsurgency is trying to separate insurgents from the population. And I think P/CVE is doing something similar in terms of trying to separate violent extremist groups from the communities where they’re recruiting and organising so that the communities will turn on them. And in the US context, looking down the street, I have violent extremist neighbours – they have the Confederate flag, they have guns, they go to rallies. And as I try to imagine, what is a P/CVE programme to engage them? I have such a hard time. It’s so much easier for me to think about Afghanistan. (Interview 7)

Albeit recognising that P/CVE and counterinsurgency have a shared objective to ‘separate insurgents from the population’, Marte admits that it is difficult to imagine such a tactic applied to white supremacist violent extremists in the US. The fact that they ‘have such a hard time’ when they ‘try to imagine’ applying P/CVE to their ‘violent extremist neighbours’ is related, in part, to what they called ‘the scale of the problem’. What happens when an extremist ideology is ‘now a fourth of your population, or a third of your population’, they wondered (Interview 7)? When violence being prevented is Islamist extremism, ‘the community’ is understood to be a Muslim community, an easily identifiable and racially marked target in need of governmental reform (Ali 2014). When the violence being prevented is white supremacist violent extremism within the US, however, ‘the community’ is the majority of the population – the community is everyone.

Thus, even with an attempt to move away from the explicitly racial markers of the Muslim community as the object of P/CVE interventions, it remains difficult if not impossible to imagine ‘the community’ as a ‘thing’ in the context of white supremacist violent extremism. What does a P/CVE programme in the US look like when the target group is white suburban residents? The P/CVE agenda’s emphasis on disciplining and reconfiguring social relationships within communities as a means of violence prevention emerges from a grammar that does not align with perceptions of non-Muslim communities in the Global North. Identifying, mapping and de-radicalising white supremacists in the Global North is incommensurate with the articulations of ‘the community’ that currently exist within P/CVE. The inability to conceptualise the implementation of P/CVE in white communities is not just a failure of imagination, but rather reveals the deep grammar of P/CVE as built on racialised notions of prevention, community and radicalisation.

## ‘Turning America’s national security tools at whole segments of the country’: the US’s disavowal of white supremacist violent extremism

The attempt to address white supremacy as a form of domestic terrorism in the US occurred following the attacks on the US Capitol on 6 January 2021. In February 2021, US Secretary of Homeland Security Alejandro N. Mayorkas proclaimed combating domestic violent extremism (DVE) as a national priority area, a first for the department. This shift came in response to the Biden administration’s pronouncement that ‘domestic terrorism is both persistent and evolving – and, according to the US Intelligence Community and law enforcement, “elevated” in the threat it now poses’ (United States 2021, 5–6). A domestic violent extremist is defined by both the DHS and FBI as

an individual based and operating primarily within the United States or its territories without direction or inspiration from a foreign terrorist group or other foreign power who seeks to further political or social goals wholly or in part through unlawful acts or force or violence. (FBI and DHS 2021, 2, n. 3)

Of the DVE threats faced by the US, ‘racially or ethnically motivated violent extremists (principally those who promote the superiority of the white race) and militia violent extremists are assessed as presenting the most persistent and lethal threats’ (United States 2021, 6). Domestic violent extremists or domestic terrorists are differentiated from home-grown violent extremists in that the latter are motivated by the ideologies of FTOs (Congressional Research Service 2021). However, as Shirin Sinnar (2021) points out, these are racialised categorisations dependent on tenuous distinctions between domestic and foreign, national and international, and which reserve the DVE label for white supremacists, anti-government or anti-authority violent extremists, and other non-Muslim actors. In short, an act of terrorism or violent extremism perpetrated in the US by an individual professing ties to Islam will always be read as foreign.

While the national threats of white supremacy and far-right extremism are nothing new in the US, the wide-reaching political attention and pressure to explicitly name such violence domestic terrorism is historically unprecedented. The ideological and political environment that has enabled white supremacist violence to continue unchecked is intertwined with a long history of domestic counterterrorism and counterinsurgency tactics to pacify Black and brown communities at home. The disavowal of white supremacist violent extremism legitimises a security state and expands surveillance and policing that continues to disproportionately target and harm communities of colour. The very racist ideologies that enabled post-9/11 policing and surveillance of racialised and minoritised communities the world over have suddenly been named the subject of counterterrorism.

Indeed, identification of DVE as the most pressing threat to the US was a dramatic change from a few years prior, when President Trump’s National Strategy for Counterterrorism proclaimed that ‘we remain a nation at war’ and ‘our principal terrorist enemies are radical Islamist terrorist groups that seek to conduct attacks globally, violate our borders and radicalize and recruit potential extremists within the United States and abroad’ (United States 2018, 1). While Trump acknowledged that domestic terrorism was ‘on the rise’, his administration consistently downplayed the threat posed by the far right and instead worked to incite his followers to radicalise towards violence. Further, while the Countering Violent Extremism (CVE) grant programme, started by the Obama administration in 2011, focused nearly exclusively on Muslim and Arab American communities, some funding was actually directed towards far-right radicalisation, funding that was later cut by the Trump administration.^5^ Trump and his team also discussed renaming the CVE programme ‘Countering Radical Islam’ or ‘Countering Violent Jihad’. As a result, Biden made a campaign promise to Muslim and Arab American communities that, if elected, he would close the CVE office, which Trump had renamed the Office for Targeted Violence and Terrorism Prevention (OTVTP). While Biden did effectively close it, in its place he created the Center for Prevention Programs and Partnerships (CP3), which, as scholars point out, is a mere rebranding of the OTVTP and retains the same flawed and exclusionary counterterrorism framework created during the Obama administration. As Harsha Panduranga writes:

By broadening its focus from Muslims to a wider spectrum of political violence and the indeterminate category of targeted violence, DHS may avoid charges of anti-Muslim bias. However, doing so simply expands the reach of the ineffective and discriminatory CVE model. (2021, 3)

Placing white supremacy, anti-government and other forms of far-right extremism at the centre of his counterterrorism agenda, Biden appears to be offering a corrective to violence and radicalisation emboldened during the Trump era. However, rather than devising new violence prevention strategies, his administration is merely repackaging the discriminatory logic of CVE and targeting, at least in theory, a newer and whiter audience.

As Andrea Miller and Lisa Bhungalia (2022) recently argued, this rhetorical move attempts to render white supremacy an abject form of whiteness, as antithetical to US liberal democracy. In other words, the explicit naming of racially or ethnically motivated violent extremism is a crucial disavowal of a particular iteration of white supremacy that interrupts the consolidation of whiteness for the US security state. It is not a disavowal of white supremacy writ large; rather, it is a disavowal of unacceptable demonstrations of white supremacy. Importantly, the disavowal of white supremacy by the US functions as an elusive yet effective strategy of upholding imperial power. Both the disavowal of white supremacy *and* white supremacy as a foundational principle of the US function as a ‘double articulation’ (Bhabha 1984, 126), which is intentionally built on ambivalence and thus serves as a way of normalising the racialised violence through which US liberalism is constructed and secured.

The inclusion of white supremacy as a target of domestic counterterrorism can thus be read as performative, enabling the solidification of whiteness ‘precisely through the performance of disavowing it’ (Miller and Bhungalia 2022, 5). The ambivalence of this disavowal presents a challenge for the coherence of the P/CVE agenda given its reliance on racialised constructions. As Ali explained:

The question I think that we’re facing right now is, are the new trends in violent extremism – are they a unifying agenda for the international community? Is there one set of policies and approaches that are useful in North America and the West when they’re dealing with right-wing violent extremism that is also relevant to the rest of the world that are dealing with different varietals? I think what we’re seeing is some differentiation in the trends in terrorism right now, and we are seeing less of a common agenda. And it’s going to make things a little bit more complicated. (Interview 1)

Ali recognised that the ‘different varietals’ raise questions about the relevance of currently existing tools. They asked, ‘How do you talk about what happened with the US Capitol in Washington, DC in the same breath [as] talking about fighters going to Ukraine? You don’t, and they’re very different answers’ (Interview 1). Here, Ali is referring to the issue of foreign fighters travelling to Ukraine to fight against the Russian occupation, a minority of whom are believed to hold far-right views and thus could pose a threat upon return to their home countries (Kaunert, MacKenzie and Léonard 2023). The emerging attention to violence enacted by white supremacists and the far right, neither a coherent nor an easily intelligible group, indeed makes things ‘a little bit more complicated’, as the performative disavowal comes into direct conflict with the mandates of projects articulated through racialised constructions of the community, prevention and radicalisation.

Consider, for instance, the US’s designation of the the Russian Imperial Movement (RIM) as a foreign terrorist organisation in 2020. This designation came as a surprise to many experts and was discussed as bearing transformative capabilities regarding the orientation of the US’s overall counterterrorism policy. Yet, as Búzás and Meier (2023) argue, RIM – ­currently the sole white supremacist organisation designated by the US as a terrorist organisation – functioned as a ‘fig leaf’ to silence opponents of the Trump administration, while also responding to the public pressure to tackle the problem of far-right extremism and violence. The choice of RIM, a relatively obscure and unknown group, thus served to rhetorically disavow white supremacy, while simultaneously minimising the risk of drawing attention to white supremacist organisations within the US. The tactic of performatively disavowing white supremacy through its inclusion in counterterrorism or P/CVE initiatives is not exclusive to the US but is also seen, for example, in the UK and Germany. As Michelle Bentley (2018) argues, the UK Prime Minister Theresa May’s approach to counterterrorism clearly names Muslim populations as the prevailing threat to ‘British values’ even while the Prevent review in 2011 made it clear that far-right extremisim would be made a priority. In addition, Meier’s (2020) analysis of government responses to white supremacist violent attacks in Germany reveals the difficulty if not improbability of disavowing white supremacy when it is a hegemonic component of German national identity. Meier rightly cautions that:

the precise construction of white supremacist power structures may differ across white-majority countries … What is comparable, however, is white supremacy’s interest in perpetuating itself, and so we should expect to observe similar dynamics to those playing out in Germany in any country where white supremacy is a hegemonic component of national identity. (2020, 9)

Nations such as the US, the UK and Germany, among others, founded on white supremacist ideals while attempting to embrace multiculturalism as core democratic values, are facing the limits of liberalism as a means to counteract or address white supremacy.

Ultimately, the particular strand of US exceptionalism and nationalism based on white supremacy that enabled the global war on terror is also, in part, responsible for the enactment of what is now being identified as white supremacist violent extremism. The last two decades have produced new iterations of political violence that have their sights trained on the very government institutions that merely three years prior applauded and sanctioned their ideologies and beliefs. The idea that the violence of white supremacy can be disrupted or prevented by the same counterterrorism architecture established during the global war on terror – a series of perpetual wars predicated on the privileges of whiteness and the dominance of the US state – is more than just illogical. The Biden administration’s naming of white supremacy as domestic violent extremism suggests a deeper impulse to correct the illiberal reign of Trump by indicating racism as an explicit enabler of violence. It is an attempt to cast out – at least rhetorically – the racism that has always been at the heart of the US political and social order. Substantively and transformatively addressing white supremacy, however, would require a much deeper interrogation of the role that whiteness plays throughout the whole of US social and political life. The constructed ambivalence – or, even more so, the illogic to the disavowal of white supremacy – stands in contrast to the foundational imaginings of P/CVE, which purports to be about all forms of violent extremism and yet is constructed through racialised discourse with objectives that include the management of presumably risky populations and states both within and outside of the Global South.

## Conclusion: far-right extremism, domestic anti-terror agendas and the recalibration of global counterterrorism

We conducted the expert interviews in spring 2021, shortly after the 6 January attack on the US Capitol and the following condemnation of white supremacy by President Biden. In the subsequent months, terrorism experts discussed the threat white supremacist violent extremism posed to Western democracies. Overall, however, the consensus among our interviewees was that they were unsure whether the current counterterrorism architecture or the P/CVE agenda had the capacity to tackle white supremacy in the United States and beyond. This led us to an analysis of what we call the ‘thingification’ of P/CVE – its ability to opportunistically shapeshift in different contexts in the service of various actors while nonetheless relying on and reproducing racialised constructs of community, prevention and radicalisation. While the Biden administration’s disavowal of white supremacist violent extremism seemed to signal a shift in the US’s orientation towards violent extremism, we suggest that the current domestic counterterrorism approach is unable to truly address white supremacism, as its pillars are rooted in a violent history of white supremacism and exclusionary logics of whiteness.

Even so, the US National Strategy on Countering Domestic Terrorism is regarded as a ‘first-of-its-kind policy document’ and has become a blueprint for a counterterrorism agenda that focuses specifically on the threat of far-right extremism. The International Centre for Counter-Terrorism, for example, released a policy brief on how to use the US National Strategy on Countering Domestic Terrorism as a model for European states and multilateral institutions. The authors recommend that the

EU counter-terrorism strategy should explicitly include far-right extremism as a current and emerging threat in its framework. While Islamist terrorism should continue to remain on the counter-terrorism agenda, the EU is behind on recognising and developing measures to specifically challenge the rise of the far-right. Individual Member State efforts need to be consolidated into a comprehensive, regional framework. (Leidig and van Mieghem 2021, 10)

The US strategy and its move towards including far-right extremism into its measures of counterterrorism has significance far beyond the US. The adaptation of similar policies in other states – mostly in the Global North – works to symbolically recalibrate global counterterrorism paradigms, while simultaneously upholding the power of whiteness.

## Notes

1 We are conscious of the risks of centring the US within a discussion of global counterterrorism. Furthermore, we are aware that global counterterrorism and P/CVE policies and programmes are not reducible to the US’s agenda and that many countries, particularly in the so-called Global South, have deftly reinterpreted the P/CVE agenda and implemented programmes that contribute to violence prevention actions in line with their peacebuilding and development objectives. Nonetheless, we maintain that the US’s domestic and foreign policy has global implications and that tracking the evolution and developments within the US is important for analysing international security regimes.

2 We recognise that the Global South/Global North binary is analytically and theoretically limited and that it projects a division and opposition of the world that does not cleanly or clearly exist. Nevertheless, given that much of P/CVE policy and progamming relies on and reproduces the South/North conceptualisation, we chose to use it in this article.

3 It is important to recognise that the origins of peacebuilding do not lie with the United Nations, for example, but rather with local initiatives including the Mindanao Peace Institute, the Nairobi Peace Initiative, and the Western African Network for Peace Building, among others. However, in this article when we refer to ‘peacebuilding’, we are primarily referring to Western-led peacebuilding paradigms.

4 All P/CVE practitioners and experts gave informed consent; however we chose to use pseudonyms to protect their identities.

5 For instance, Life After Hate, a group which offers exit programmes and intervention services to white supremacists, were slotted to receive a 400,000 USD CVE grant, which was then cancelled by Trump-appointed US Homeland Security Secretary John Kelly.
